# Oral administration of heat-inactivated *Escherichia coli* during suckling alleviated *Salmonella typhimurium*-derived intestinal injury after rat weaning

**DOI:** 10.3389/fimmu.2023.1119747

**Published:** 2023-04-05

**Authors:** Minghui Cui, Guangfu Tang, Fang Yan, Shunshan Wang, Xi Wang, Junhu Yao, Xiurong Xu

**Affiliations:** College of Animal Science and Technology, Northwest A&F University, Yangling, China

**Keywords:** heat-inactivated *Escherichia coli*, *Salmonella typhimurium*, trained immunity, postweaning diarrhea, intestinal microbiota

## Abstract

**Introduction:**

Newly weaned animals are susceptible to a wide range of microbial infections taking a high risk of developing post-weaning diarrhea. Trained immunity is the capacity of the innate immune system to produce a stronger and non-specific response against a secondary infection after the inflammatory response caused by previous stimulus has returned to normal state. The objective of this study was to evaluate if the heat-inactivated *Escherichia coli* (IEC) as an immunostimulant on suckling pups elicits a protective effect on the intestine of post-weaning rats challenged with *Salmonella Typhimurium* (*S.Typhimurium*). We adapted a newborn rat model for this purpose.

**Methods:**

Sixty newborn pups were randomly separated into two groups: IEC group (n =30) orally administrated IEC during suckling, while the CON group received orally the same dose of saline. Both of the two group challenged with various doses of *S.Typhimurium* after experiencing a 4-week resting period. Twelve of individuals were selected to detect the survival rate, and ten of the rest were necropsied 48 hours post-challenge.

**Results and Discussion:**

The results showed that oral administration of IEC during suckling alleviated the injury in ileal morphology induced by post-weaning *S.Typhimurium* infection via increasing the levels of two tight junction proteins [zonula occluden-1 (ZO-1) and Occludin-1] and several secreted proteins (Lysozyme, Mucin-2, and SIgA) in the intestinal mucosa. Furthermore, the pre-stimulation with IEC significantly increased cytokines tumor necrosis factor-alpha (TNF- α) and interleukin-1 beta (IL-1 β) expressions in an enhanced secondary reaction way after experiencing a 4-week resting period. This implicated the possible involvement of trained immunity. The 16S rDNA sequence results showed that pre-stimulation with IEC decreased the abundance of *Clostridia, Prevotella, Christensenellaceae_R-7_group* and *Parabacteroides* after intestinal infection of *S.Typhimurium*. Our results confirmed that the previous oral administration of IEC had a protective effect on *S.Typhimurium*-induced intestinal injury in weaned rats by inducing a robust immune response. The present study suggested a new strategy for preventing intestinal infection of newborn animals.

## Introduction

Weaning is when the young mammals ceased from mother’s breast feeding, which often leads to severe stress that results in weakened disease resistance and subsequent diarrhea (1). Post-weaning diarrhea is the main disease of mammals, and severe diarrhea adversely affects the development of newly weaned animals and their product performance as an adult (2–4). One of the major causes of diarrhea is intestinal infection, especially unpredictable and complex bacterial or viral infections. Due to the lack of protection provided by breast milk and the immature state of adaptive immunity, newly weaned animals are more susceptible to a wide range of pathogenic infections (5).

The classical prevention strategy for infection-induced diarrhea in humans and animals relies on immune memory, which has been defined to utilize adaptive immunity that requires T and B lymphocyte functioning (6). Interestingly, increasing evidence suggests that faster and stronger protective mechanisms against reinfection also exist in plants and invertebrates that lack adaptive immune systems, which is a de facto immune memory behavior (7, 8). Furthermore, similar phenomena have been detected in epidemiological studies in vertebrate models, whose innate immune cells produce a non-specific immune response when confronted with adverse challenges and respond to secondary infections more strongly (9–14). This enhanced memory response of innate immunity to later stimuli is defined as “trained immunity” (15). Predictably, this emerging field of immunology provides a new theoretical direction for preventing the infection of unpredictable pathogens.

Current studies demonstrate the potential of training innate immunity during suckling to prevent post-weaning diarrhea in mammalians. Some live attenuated vaccines were proven effective in inducing trained immunity that responds to heterologous challenges (16, 17). Bacillus Calmette-Guérin (BCG) is the most commonly used live attenuated vaccine in training immunization models. A recent study has shown that 5- to 7-day-old neonatal mice intraperitoneally injected with BCG and bacterial lipoprotein (BLP) were conferred protection against polymicrobial sepsis through trained immunity (18). However, oral vaccination with live BCG led to its detection in the lymphoid organs a few days after immunization (19). Thus, using inactivated immunostimulants seems preferable since it increases the security of stimulation. *Escherichia coli* (*E. coli*) is one of the primary pathogens that cause intestinal infection. Therefore, using the inactivated *E.coli* as the immunostimulant might induce not only the innate immunity in suckling animals to deal with unknown-pathogen infection after weaning, but also strengthen the adaptive immunity against the possible infection of *E. coli*.

Based on the above, the objective of this study was to evaluate whether heat-inactivated *Escherichia coli* (IEC) as an immunostimulant on newborn pups elicits a protective effect on intestinal injury in post-weaning rats, which were challenged with *S.Typhimurium*, another primary pathogen that can cause intestinal infection. To examine whether the innate immune in the intestine was trained and involved in protecting rats against the intestinal *S.Typhimurium* challenge, we also investigated the immune-related indicators in the three periods of the classical trained immune model related to the innate immune in the intestine. Our study may provide a new strategy for preventing intestinal infection in newly weaned animals.

## Materials and methods

### Heat-inactivated *Escherichia coli* (IEC)

The IEC solution consisted of approximately 10^10^ colony-forming units (CFU) of IEC (*E.coli* K88, gifted from Prof. Huping Xue, Northwest A&F University, Xianyang, China) in sterile saline (NS). Heat inactivation of *E.coli* was performed for 40 minutes at 70°C, which was prepared following the protocol described (20), with the exception of a shortened inactivation time. After inactivation, 100 μl of bacterial culture was coated on LB solid medium (Land Bridge Co., Ltd., Beijing, China). The same volume of NS and live *E.coli* were the negative control and the positive control, respectively, to evaluate whether IEC was successfully inactivated.

### Animals and experimental design

Six adult Sprague-Dawley rat breeders were purchased from the Chengdu Dashuo Laboratory Animal Technology Co. Ltd. (Chengdu, China). Rats were housed under a controlled environment with access to food and water ad libitum throughout the feeding experiment.

**Figure 1:**
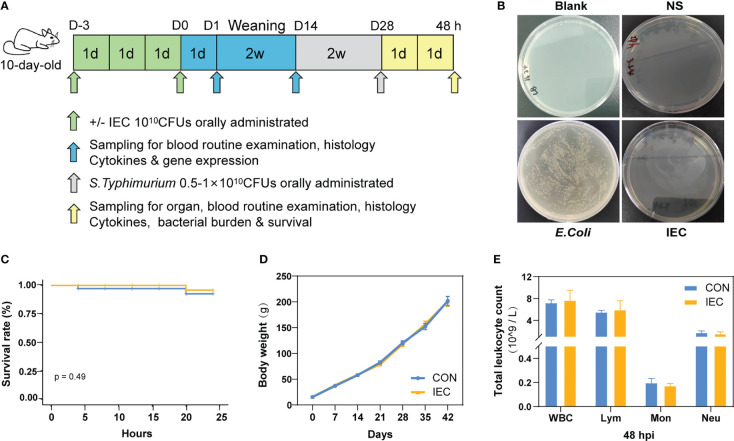
Study design and protective effect of IEC against *Salmonella* infections. **(A)** Schematic diagram of the experimental procedure. **(B)** IEC inactivation effect test. **(C)** Rats survival rate was recorded for 24 hours *Salmonella* infection (n =12/group). **(D)** The body weights of rats during the whole experiment (n = 10~18/group). **(E)** Counts of leukocyte were detected by blood routine examination at 48 hpi (n = 10/group). The data are presented as mean ± SEM. WBC, white blood cell; Lym, lymphocyte; Mon, monocyte; Neu, neutrophilicgranulocyte; hpi, hours post infection.

Within 5 days after farrowing, each litter of the ten pups was randomly assigned based on body weight into two experimental groups (CON group and IEC group), and each pup was given its own number for identification. At 7 and 10 days of age (D-3 and D0), each suckling pup (mixed sex) in the IEC group received 1 ml IEC to induce first stimulation, while that in the CON group received 1 ml NS (**Figure 1A**). After weaning, each pup was reared into a single cage for subsequent challenge with *S.Typhimurium* on two weeks after weaning (4 weeks after the first stimulation). All the pups in the experiment were received the same batch inoculum of IEC and *S.Typhimurium*.

### Challenge

The *S.Typhimurium* CVCC542 (Gift from Prof. Huping Xue, Northwest A&F University, Xianyang, China) was cultured overnight at 37°C in LB liquid medium (Land Bridge Co., Ltd., Beijing, China) and then centrifugation at 5,000 rpm for 10 min. After being washed and suspended in 0.9% NS, the bacterial suspension was adjusted to the desired concentration according to a standard optical density curve. Considering that rats might die rapidly after challenged with high doses of *salmonella*, the rats were orally administrated with various doses of *S.Typhimurium* to challenge after a gradient test in our research. The suspensions containing 5×10^9^ CFU/ml and 1×10^10^ CFU/ml of *S.Typhimurium* were used for the sample collection and survival rate experiment, respectively. Twenty-two of rats in each group were challenged, twelve of which were selected to detect the survival rate, and ten of the rest were selected to record the body weight, monitor the clinical symptoms at different stages, and collect the biological samples for subsequent investigation. The body weight, survival rate, and organ index were evaluated as described previously (21, 22).

### Sampling

On day 1 and day 14 after oral stimulation of IEC, four pups were randomly chosen from each group, and the blood samples were collected into tubes with anticoagulant (EDTA) when the rats were in an anesthetic state. Rats were then euthanized. Except for the ileum tissue sections, the remaining ileal tissue and mucosa were placed in sterile, RNase-free optimized tubes and immediately frozen in liquid nitrogen after collection, and then stored at -80°C for total RNA isolation and concentration determination of several proteins by ELISA. The quantity and quality of the extracted RNA were assessed using an NanoDrop 2000 UV-vis spectrophotometer (Gene Company Limited, China) and agarose gel electrophoresis (1%), respectively. Fourty-eight hours after the intragastric challenge of *S.Typhimurium*, samples were also collected as mentioned above, the blood, liver, and spleen were also sampled under the sterile condition as soon as possible for *Salmonella* enumeration additionally.

### Histopathology

To prepare samples for the observation of ileum histopathology, the ileum tissues were dipped in 10% neutral buffered formalin (Solarbio Co., Ltd., Beijing, China) and made paraffin sectioning for histopathological analysis (23). The intestinal villus height, crypt depth, and the rate of villus height to crypt depth were calculated by ImageJ software as indicators of intestinal damage and histological inflammation.

### Determination of *Salmonella* colonies

The colonies of *Salmonella* in organs were first assessed using a culture-dependent approach. The samples, including blood, liver and spleen, which were aseptically collected from the euthanized rats at 48 hours post-infection (hpi), and then weighted and homogenized in NS. The suspension from organ homogenates or the blood samples was serially diluted and cultured on DHL agar (Land Bridge Co., Ltd., Beijing, China) plates. The plates were incubated under the condition at 37°C for 24 h and checked for *Salmonella* colonies.

The colonies of *Salmonella* in organs were also determined by absolute quantitative polymerase chain reaction (qPCR). The method in detail was followed as described previously to measure counts of total bacteria and of *S.Typhimurium* (24). Primers for our assay are presented in **Table 1**.

**Table 1:** Primers sequence table.

Target gene	Primer	Primer sequence(5′-3′)	Source
Total bacteria	Forward	ACTCCTACGGGAGGCAGCAG	(25)
	Reverse	ATTACCGCGGCTGCTGG	
*Salmonella Typhimurium*	Forward	TACAGGTGACTGCGGGCTTATC	(26)
	Reverse	CTTACCGGGCAATACACTCACTA	

### Measurement of leukocytic parameters

The blood samples with anticoagulant were used for leukocytic parameters, including differential WBC count. They were measured by an auto hematology analyzer (BC-2800Vet, Mindray Medical Co., Ltd., China).

### RNA isolation and quantitative real-time PCR

Total RNA of ileum mucosa samples was extracted using the Trizol reagent (Invitrogen, Carlsbad, CA, USA) and reverse transcribed to cDNA with Evo M-MLV RT Premix for qPCR AG11728 (Accurate Biotechnology (Hunan) Co., Ltd, China) after quantified the concentration and purity of total RNA. Gene expression was detected using the Power SYBR™ Green PCR Master Mix (Thermo-Fisher Scientific) on the LightCycler^®^ 480 Instrument II (Roche Life Science). The primer pairs encoded Occuldin-1, ZO-1, MUC2, LYZ, TNF-α, IL-1β, and IL-6 were presented in **Table 2**. RT-qPCR was performed with a volume of 10 μl containing 5 μl SYBR Green Mix (innovagene, Hunan, China), 0.5 μl each of forward and reverse primers, and 5 μl of cDNA as template transcribed from a standardized amount of total RNA. The qPCR conditions were an initial denaturation step at 95°C for 30 s, 40 cycles at 95°C for 10 s, and 60°C for the 30s followed. Melting curve was programmed to be 95°C for 10 s, 65°C for 60s followed by 1 s for 97°C. The 2^-ΔΔCt^ method was used to calculate relative gene expression levels with GAPDH as an internal reference between different samples.

**Table 2:** The primers of the investigated genes for RT-qPCR analysis.

Target gene	Primer	Primer sequence(5′-3′)	Source
GAPDH	Forward	GGCACAGTCAAGGCTGAGAATG	(27)
	Reverse	ATGGTGGTGAAGACGCCAGTA	
TNF-α	Forward	ACCTCCTCTCTGCCATCAAG	(28)
	Reverse	CTGAGTCGGTCACCCTTCTC	
IL-6	Forward	GTCAACTCCATCTGCCCTTCAG	(29)
	Reverse	GGCAGTGGCTGTCAACAACAT	
IL-1β	Forward	AATGCCTCGTGCTGTCTGA	(27)
	Reverse	GGATTTTGTCGTTGCTTGTCTC	
ZO-1	Forward	AAGCCAGTCACGATCTCCCG	(30)
	Reverse	GCGCTCTTCCTCTCTGCTCC	
Occludin-1	Forward	CAACGGCAAAGTGAATGGCA	(31)
	Reverse	CTTTCCCCTTCGTGGGAGTC	
Lysozyme (LYZ)	Forward	CAAGCCATACAATGTGCGAAGAGAG	(32)
	Reverse	TGTTGGTTTGAGGGGAAAGCAAG	
MUC2	Forward	CTGAGGAAGGCCAAGTTTAC	(33)
	Reverse	CAGGTCCCAGAGAGGAAATA	

### ELISA

The concentration of SIgA, LYZ and MUC2 in ileal mucosa, which was collected at 48 hpi, were measured using the rat SIgA ELISA kit, rat LYZ ELISA kit, and rat MUC2 ELISA kit (Mlbio, Shanghai, China), respectively, following the instructions of the manufacturer.

### DNA extraction from the samples

Cetyl trimethyl ammonium bromide (CTAB) was used to extract the microbial DNA from samples (34). The quantity and quality of the extracted DNA were assessed using an ND2000C UV-vis spectrophotometer (Gene Company Limited, China) and agarose gel electrophoresis (1%), respectively.

### Microbiota sequencing, sequence processing and analysis

All DNA samples were diluted with the Tris-EDTA solution, and then the diluted DNA was used to amplify the V4 hypervariable region in bacterial 16S rRNA gene using the forward primer 515F (5’-GTGCCAGCMGCCGCGGTAA-3’) and the reverse primer 806R (5’-GGACTACHVGGGTWTCTAAT-3’). The amplicons were separated on 2% agarose gels and further quantified using the Qiangen Gel Extraction Kit (Cat No. DP209) (Qiagen Sciences Inc., Germantown, MD) and quantified using QuantiFluor™-ST (Promega, USA) according to the manufacturer’s protocol. Finally, the libraries were sequenced on an Illumina NovaSeq6000 platform (Beijing Nuohezhiyuan Technology Co.LTD., Beijing, China), without the negative control sequencing.

All sequences were analyzed using the following procedures. Based on the unique barcode, all reads were truncated by cutting off the barcode and primer sequence. Then, contiguous DNA sequences were assembled using FLASH software (V1.2.11, http://ccb.jhu.edu/software/FLASH/), the original Tags data (Raw Tags). Raw tags were quality-filtered and processed using fastp (Version 0.20.0) software, and then the chimeric sequences were identified and removed using the QIIME2 DATA2 plugin to obtain the feature table of amplicon sequence variant (ASV). According to the SILVA database (version 138), the ASV in each sample was assigned to corresponding taxonomies at phylum, class, order, family, and genus, respectively. Alpha diversity was mainly measured *via* Chao1 index to analyze the richness and uniformity of different microbial communities in the sample. The beta diversity of the microbiome was displayed by a principal coordinate analysis (PCoA), which was conducted based on the Bray-Curtis distance using QIIME2. Wilcoxon rank-sum test analysis was used to determine the significantly different species at each taxonomic level.

### Statistical analysis

All data were processed preliminarily using Microsoft Excel 2016 before any statistical analyses were conducted. Survival data were compared by log-rank test. The data were analyzed for the homogeneity of variances and normality using Levene’s and Shapiro-Wilk’s tests, respectively. Data of two samples with normal distribution were compared by Student’s t-test. Wilcoxon rank-sum test was used for comparing the heterogeneous or non-normal distribution data. Statistical Product and Service Solutions (SPSS, Inc, Chicago, IL, USA) was used for all statistical analyses. The figures for data visualization were performed using GraphPad Prism 8.0 (GraphPad Software Inc., San Diego, CA). The data were presented as mean ± SEM. A *P < 0.05* was considered as statistically significant, and *P-*values between 0.05 and 0.10 represent a statistical trend. In figures in our results section, the asterisks denoted statistical significance (*P < 0.05; **P < 0.01; ***P < 0.001).

## Result

### Effect of IEC initial stimulation on the survival rate, growth status and leukocyte parameters

We first tested whether IEC was completely inactivated upon heat treatment. As shown in **Figure 1B**, no *E.coli* grew in the plates of blank control, saline control and IEC groups, suggesting that IEC was completely inactivated. The following experiment was designed to explore the effect of oral immunization with IEC on reducing *Salmonella* infection in the post-weaning rat. The results in **Figure 1C** suggested no statistical difference in survival rate between CON and IEC groups within 24 hours of the challenge (*P* > 0.05), however the CON group rats showed more depressed spirit and disorganized fur of body.

As shown in **Figure 1D**, there was no difference in body weight between the two groups during the experiment. No significant differences were observed in the leukocyte-related parameters at 48 hpi between the two infection groups (**Figure 1E**).

### Organ index analysis and bacterial colonization

**Figure 2:**
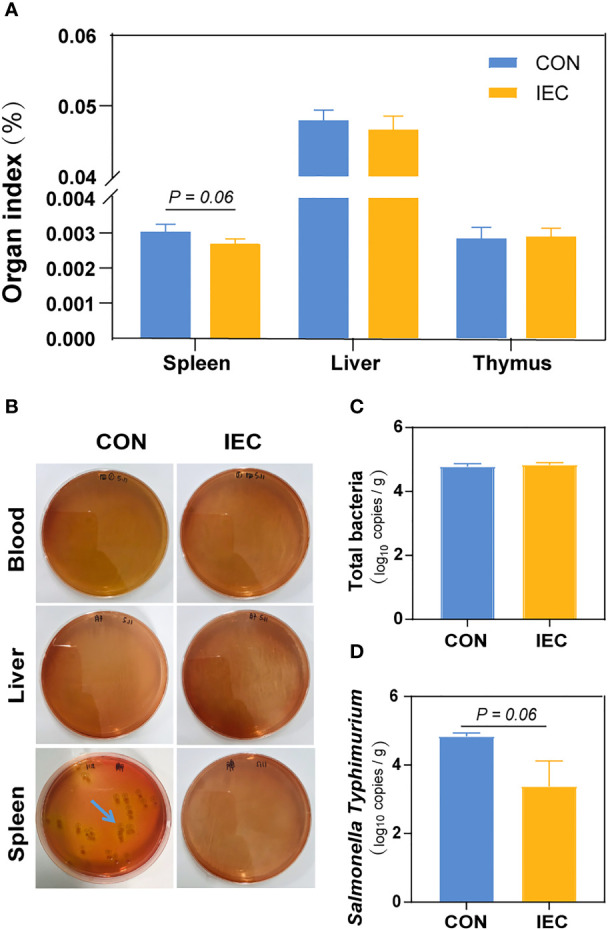
IEC pre-stimulation provided protection against *Salmonella* infections. **(A)** Organ indexes analysis of the spleen, thymus and liver of rats in the groups (n =10/group) at 48 hpi. **(B)** Bacterial burdens in organs and blood at 48 hpi were determined *via* DHL plates (n = 5/group). **(C, D)** Number of total bacteria and *S.Typhimurium* in spleen of rats (n =10/group) at 48 hpi. The data are presented as mean ± SEM. hpi, hours post infection.

To determine the extent of *Salmonella* infection, loads of *S.Typhimurium* in the blood, liver and spleen were tested. Compared with the CON, pre-stimulation with the IEC did not affect the organ index of the thymus and liver, except that the spleen weight of rats pre-treated with IEC showed a reducing tendency (*P* = 0.06) (**Figure 2A**). *S.Typhimurium* at 48 hpi was not detected in the blood and liver samples from both groups *via* a culture-dependent approach, but the spleen tissues of the CON rats had more colony counts than those of the IEC rats (**Figure 2B**). Consistent with the result of colony growth, *S.Typhimurium* quantitated by qPCR in spleens of the IEC rats had a reducing tendency at 48 hpi (*P* = 0.06) (**Figure 2D**). However, the total bacterial count in the spleen was not significantly different between the two groups (**Figure 2C**).

### Pathological and intestinal barrier function analysis of ileum tissues

**Figure 3:**
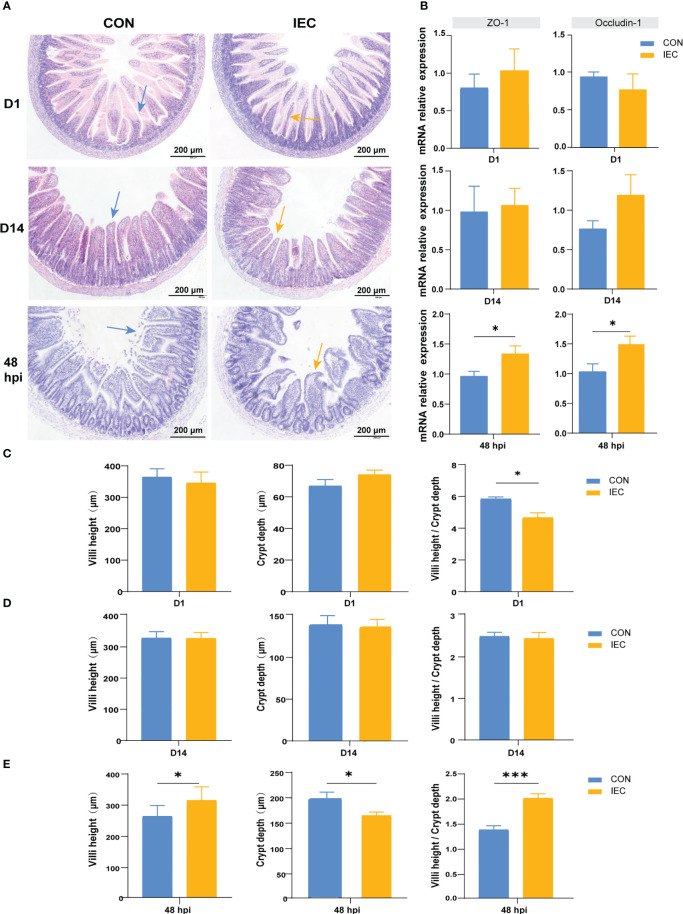
Pathological, changes of intestinal villus and crypt and the relative genes expression in two groups in different period. **(A)** Intestinal morphology shown of the ileum tissues of rat in different period (n = 4~10/group). **(B)** The gene expression of intestinal barrier function (n = 4~10/group). **(C-E)** Histological evaluation of ileum between two groups of rats in different period (n = 4~10/group). The data are presented as mean ± SEM. Asterisks indicate significant difference between the IEC group and the CON group (*P < 0.05; ***P < 0.001). *ZO-1*, Zonula occluden-1.

Our study aimed to investigate whether IEC pre-stimulation on neonatal pups during suckling could alleviate intestinal injury caused by *Salmonella* infection in post-weaning rats, therefore the changes in the ileal histological morphology were first observed and measured after *Salmonella* infection. As shown in **Figure 3A**, oral administration of IEC did not change the typical appearance of the intestinal structure and the gene expression of intestinal barrier proteins ZO-1 and Occludin-1 on D1 (**Figure 3B**). However, it significantly reduced the ratio of villus height to crypt depth (VCR) (*P* < 0.05) (**Figure 3C**). Furthermore, we found better structural integrity of the ileum on D14 than on D1 in both groups (**Figure 3A**), and there were no significant differences in villus, crypt morphology and gene expression of the two tight junction proteins (**Figures 3B, D**). Intestinal structure damage was observed in the ileum of all rats at 48 hpi when compared to rats on D14 (**Figure 3A**). However, the villus height (*P* < 0.05) and the VCR (*P* < 0.001) in the IEC group were significantly higher, and the crypt depth was significantly lower (*P* < 0.05) than that in the CON group at 48 hpi (**Figure 3E**). Meanwhile, Pre-stimulation significantly increased the mRNA levels of ZO-1 and Occludin-1 compared with the CON group at 48 hpi (*P* < 0.05) (**Figure 3B**).

### Detection of immunological factors in the ileum

**Figure 4:**
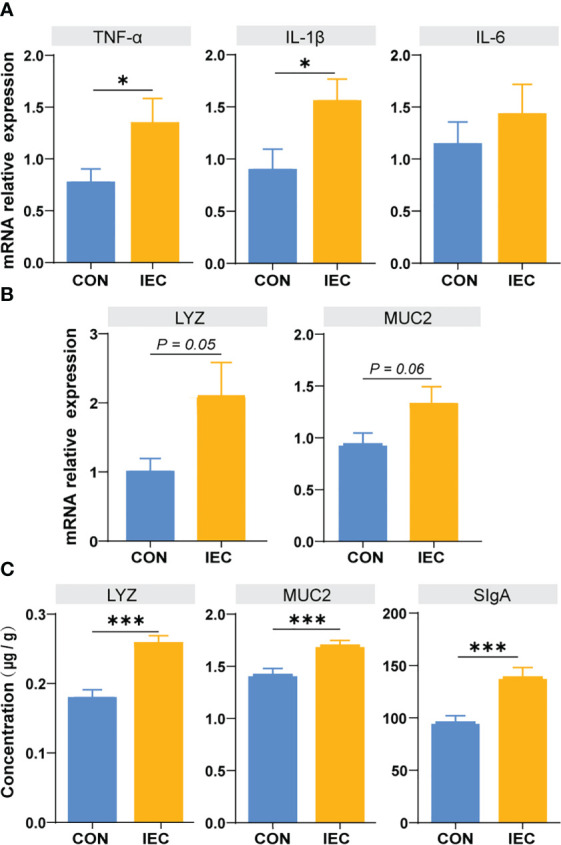
Determination of immune function factors in ileum of rats at 48 hpi. **(A, B)** The contents of TNF-α, IL-6, IL-1β, LYZ and MUC2 in ileum issue were measured by qPCR to evaluate the degree of immune response (n =10/group). **(C)** The contents of LYZ, MUC2 and SIgA in ileum musoca were measured by ELISA to evaluate the degree of immune response (n =10/group). The data are presented as mean ± SEM. Asterisks indicate significant difference between the IEC group and the CON group (*P < 0.05; ***P < 0.001). *TNF-a*, Tumor necrosis factor-a; *IL*, Interleukin; *LYZ*, Lysozyme; *MUC*, Mucin; *SIgA*, secretory immunoglobulin A; hpi, hours post infection.

To further estimate the immunological effect of pre-treatment with IEC, we detected the relative mRNA levels of TNF-a, IL-1β, IL-6, LYZ and MUC2 in the ileum issue from the CON and IEC groups after *S.Typhimurium* infection. As presented in **Figure 4**, higher expressive levels of TNF-a and IL-1β were found in IEC rats (*P* < 0.05) (**Figure 4A**), and the expression of LYZ and MUC2 tended to enhance in the IEC group (*P* = 0.05 and *P* = 0.06) (**Figure 4B**). The concentrations of LYZ (*P* < 0.001), MUC2 (*P* < 0.001), and sIgA (*P* < 0.001) in ileal mucosa were also significantly higher in IEC rats (**Figure 4C**).

### Oral administration with IEC-induced immune memory

**Figure 5:**
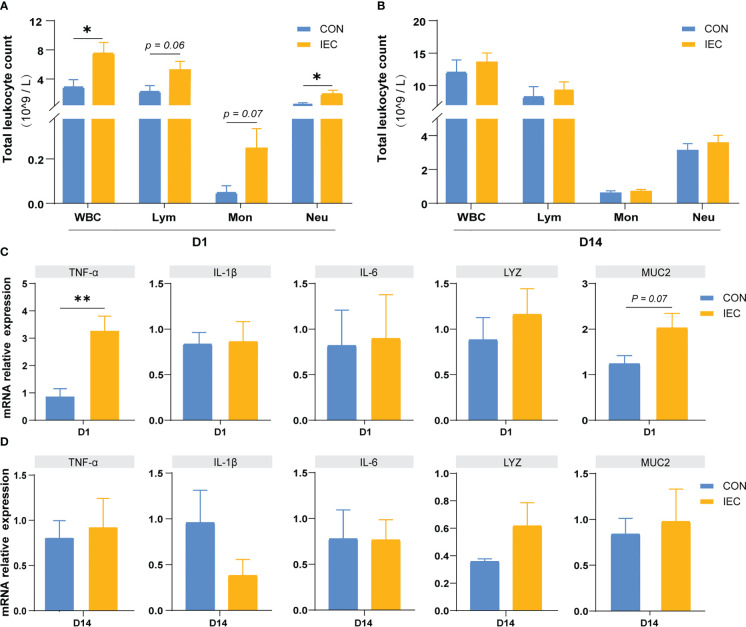
Oral administration with IEC induced immune memory. **(A, C)** Leukocytic index and gene expressions of ileum of rats on D1 (n =4/group). **(B, D)** Leukocytic index and gene expressions of ileum of rats on D14 (n =4/group). The data are presented as mean ± SEM. Asterisks indicate significant difference between the IEC group and the CON group (*P < 0.05; **P < 0.01). WBC, white blood cell; Lym, lymphocyte; Mon, monocyte; Neu, neutrophilicgranulocyte; *TNF-a*, Tumor necrosis factor-a; *IL*, Interleukin; *LYZ*, Lysozyme; *MUC*, Mucin.

To assess whether the IEC-induced alleviation of the injury caused by *S. Typhimurium* infection was involved in the immune memory or just a result of the persistent immunological activation, we measured the immune response after immunization of IEC on D1 and D14. In immune response to oral administration of IEC, the WBC and neutrophil count showed a significant increase on D1 (*P* < 0.05) (**Figure 5A**), but there were no differences in the counts of these two types of cells between the two groups on D14 (**Figure 5B**). The counts of lymphocyte and monocyte showed the same trend (*P* = 0.06 and *P* = 0.07). Likewise, levels of TNF-α (*P* < 0.01) and MUC2 (*P =* 0.07) in the ileum increased at D1 (**Figure 5C**) but completely recovered to the normal level at D14 (**Figure 5D**).

### Effect of pre-stimulation with oral administration of IEC on the intestinal microflora

**Figure 6:**
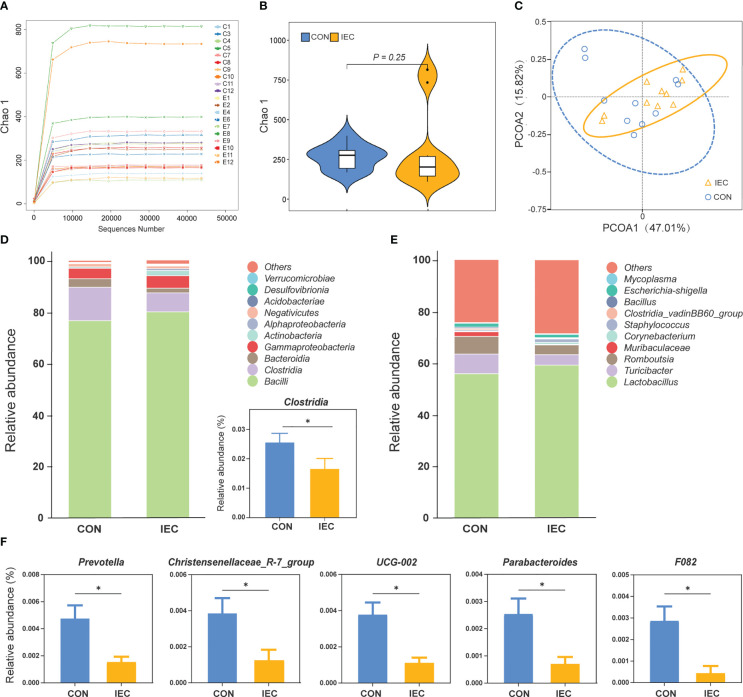
16S rRNA sequence analysis of ileal contents in rats and the microflora data diversity analysis (n =10/group) at 48 hpi. **(A)** Rarefaction curve of ileum flora. **(B)** Chao-1 Diversity Index analysis of ileum flora between the two groups. **(C)** PCoA diagram of Beta diversity in ileum flora between the two groups. **(D)** and **(E)** Relative abundance of species at class and genus level of ileal flora between the two groups. **(F)** Differential species analysis of ileum flora at the genus level between the two groups. The data are presented as mean ± SEM. Asterisks indicate significant difference between the IEC group and the CON group (*P < 0.05). hpi, hours post infection.

The effects of oral administration with IEC on the intestinal microflora communities in rats after being infected with *Salmonella* were estimated by 16S rDNA sequence. The rarefaction curves showed that nearly all the bacteria species were sequenced in the contents of the ileum of rats (**Figure 6A**). The sequencing results showed no differences in the bacterial species richness by alpha diversity analysis and PCoA of beta diversity analysis (**Figures 6B, C**). The two groups had similar bacterial distribution at the genus level (**Figure 6E**). But the relative abundance of *Clostridia* was significantly increased in the CON group at the class level (*P* < 0.05) (**Figure 6D**). Moreover, the abundance of *Prevotella*, *Christensenellaceae_R-7_group*, *UCG-002*, *Parabacteroides*, and *F082* in the CON group was significantly higher than that in the IEC group (*P* < 0.05) (**Figure 6F**).

## Discussion

Neonatal weaning increases the risk of intestinal infection, which often leads to severe diarrhea. The pathogens commonly incriminated in neonatal enteric infections include viral, protozoal and bacterial pathogens (such as *E.coli* and *Salmonella* spp.). The complexity of pathogens brings great difficulties in preventing intestinal infection, a challenge that highlights the urgent need to develop broad-spectrum protection. In this study, we investigated the potential protective effect of orally administrated IEC on intestinal injury in newly weaned rats. Our results showed that early antigenic stimulation induced a trained immunity-like phenotype in the intestinal tissue of rats. The initial stimulation before weaning improved the ability of the intestinal immune response to heterologous post-weaning infection, thus alleviating the intestinal damage caused by *Salmonella* infection. No known previous studies have shown that inactivated bacterial stimulation in suckling rats had a non-specific protective effect against secondary heterologous intestinal infections.

Based on previous research (35, 36), the present study selected heat-killed *E.coli*, the most common intestinal pathogen, as the initial stimulus, and the hypothesis was tested according to the classic model of trained immunity. Briefly, the host produced an inflammatory response after the initial stimulation, then gradually returned to the normal state and entered the resting period. When stimulated again with homologous or heterologous pathogens during this resting state, the host’s innate immune system can produce a stronger immune response to effectively deal with the re-infection (37).

Firstly, we examined the changes of relevant indicators in pups after 24 h of oral stimulation with IEC. We found that IEC-stimulated pups showed an increased expression of TNF-α and MUC2 genes in the ileum, which indicated an activated immune response. Consistent with other studies on trained immunity, the count of WBC, lymphocyte and neutrophil of the IEC pups increased compared with the CON group (38). These increases illustrated that the pups had a boosted immune response in the ileum after IEC stimulation, indicating that the first stimulation stage was successfully constructed.

It was reported that mice trained 5 weeks earlier were protected from intraperitoneal bacterial infections (39). Hence, the second infection with *S.Typhimurium* was performed two weeks after weaning (four weeks post-initial stimulation) to simulate the model of post-weaning intestinal infection. Intestinal *S.Typhimurium* infection often induces intestinal inflammations and injures the intestinal barrier (40), especially in the distal ileum (41). Therefore, our subsequent investigation focused on the changes in the ileum. The ileal histopathology, including the length of villi, the crypt depth and their ratio, is a vital index to judge the degree of intestinal damage. According to our histopathological observation, the pre-stimulation of IEC during suckling mitigated the severe injury in the ileal villi structure caused by *S.Typhimurium* infection after weaning, characterized by a decrease in crypt depth and an increase in villi height and VCR. Tight junction proteins are the important bridge between intestinal epithelial cells and are essential to intestinal barrier function, while *Salmonella* can damage this bridge by inhibiting the expression of tight junction protein genes (42, 43). We found here that the ileal injury caused by *S.Typhimurium* infection was alleviated by pre-stimulation with IEC during suckling, and this alleviation was consistent with the enhanced expression of tight junction genes ZO-1 and Occludin-1. As known, if *Salmonella* damages the intestinal barrier, it will translocate into vital organs and ultimately reach the spleen and liver *via* the infectious pathway (41). Therefore, we furtherly measured organ index and bacterial load to determine the extent of *Salmonella* infection. We found that the protective effect of pre-stimulation with IEC on intestinal structure also reduced the burden of *S.Typhimurium* colonized in the liver and spleen. A conclusion could be drawn from the above that stimulation induced by oral administration of IEC during suckling could protect post-weaning rats from the *S.Typhimurium* challenge. This protection was suggested as related to diminishing the intestinal injury.

To determine whether the protective effect induced by IEC was involved in the enhanced innate immune response in the intestine, we then examined the expression levels of cytokines in ileal tissue. TNF-α is critical for effective antibacterial host defense by activating immune cells, and IL-1β responded by monocytes and DCs in the gut, owing to its role in inflammatory cell recruitment, is considered to enhance gut protection (42). These two cytokines have been generally used as the classic markers that indicate the occurrence of trained immunity (43). In the present study, we found that the immune response in the intestine of rats in the IEC group heightened by expressing more product of TNF-α and IL-1β when encountering the secondary infection of *S.Typhimurium*, which was consistent with previous studies about trained immunity (44, 45).

The expression of Lysozyme (LYZ) and MUC2 in the ileum was also measured to investigate whether the intestinal mucosal innate immune response to re-infection was enhanced by pre-stimulation. Lysozyme in the intestine is primarily produced by Paneth cells, but it is also synthesized in neutrophils and macrophages (46). Its primary function is the bacteriolytic effect, which is an indispensable part of the gut’s innate immune system (47). The level of MUC2, produced by goblet cells, also indicates the status of intestinal mucosal immunity (48). As hypothesized, pre-stimulation with IEC increased the expression of LYZ and MUC2 at both mRNA and protein levels in the ileum after re-infection with *S.Typhimurium*, suggesting that oral administration of IEC could enhance the function of ileal mucosal innate immunity in response to secondary intestinal infections. In addition, the secretion of sIgA in the ileal mucosa was also significantly promoted after re-infection in the IEC group, which also activated mucosal immunity. In conclusion, pre-stimulation with IEC during suckling presented an augmentation in both immune response and antimicrobial capability, thereby conferring stronger protection against heterologous infection in post-weaning rats, in agreement with previous reports (18). Notably, this enhanced immune response was the consequence of a synergistic effect of both innate and adaptive immunity (49).

Another key feature of trained immunity is the return to a resting state post-training, prior to a secondary challenge (50). To exclude the possibility that the previous exposure to IEC resulted in a sustained response, an extra time point on D14, the day between IEC stimulation and *S.Typhimurium* infection, was selected for determining the immune state in the ileum before re-infection. We tested the same indicators as D1, and the results showed no difference between the two groups, demonstrating that the host response to the first stimulation has recovered to a baseline level. Therefore, it could be concluded that stimulation induced by oral administration of IEC established a trained-immunity-like phenotype in the intestine of suckling rats. Further investigations will be necessary to demonstrate whether epigenetic and metabolic reprogramming that results in trained immunity (51, 52) was involved in the enhanced innate immune response to reinfection in the gut, especially in intestinal epithelial cells.

To investigate whether oral stimulation with IEC affected the structure of the intestinal flora after *Salmonella* infection, we also detected the ileal flora diversity. We found that the oral pre-stimulation with IEC did not sharply influence the intestinal microbiota structure after *Salmonella* infection. However, the IEC-stimulated rats had a remarkable decline in the relative abundance of some microbial genera, including *Clostridia*, *Prevotella*, *Christensenellaceae_R-7_group*, and *Parabacteroides. Clostridia* can be known for their protective role in gut health (53), but increasing studies found that *Clostridia* colonization could lead to metabolic changes in the microbiota, consequently exacerbating intestinal inflammation (54). Likewise, it has been reported that the abundance of *Prevotella*, *Christensenellaceae_R-7_group* and *Parabacteroides* increased in unhealthy conditions (55–57). Conversely, the abundances of the above bacteria were prominently reduced after infection when the host was pre-stimulated with IEC during suckling, which agree with our assumption that oral administration of IEC had a beneficial effect on the alteration of ileal flora structure in rats after weaning.

Nevertheless, some remaining points should be further studied. Here we only demonstrated that intestinal immune stimulation during suckling could alleviate the heterogeneous intestinal infection after weaning through the phenotype obtained. The exact mechanisms underlying trained immunity and how it provides non-specific protective effects through IEC-stimulation are ongoing questions. Meanwhile, oral administration with IEC caused mild intestinal stress in pups, which probably affected the intestine’s absorption function in pups. Further studies will be necessary to demonstrate whether reducing the dose of the initial stimulus can produce a similar protective effect. Our findings proved that oral administration with IEC to suckling pups significantly alleviated the intestinal injury caused by *S.Typhimurium* infection after weaning.

## Conclusions

In summary, we proved that oral administration with IEC during suckling improved intestinal resistance to *S.Typhimurium* infection in the weaned rat. The evidence was characterized by stronger inflammatory response due to enhanced inflammatory cytokine and higher antimicrobial capacity in the gut innate immune system, suggesting possible involvement of trained immunity. The present study suggested a heterologous protective effect of pre-stimulation with some immunostimulants against intestinal pathogens, opening new avenues for further research on non-specific protection against unpredictable infection in intestine.

## Data availability statement

The original contributions presented in the study are publicly available. The raw data is available here: Doi: 10.6084/m9.figshare.21587829. Changes of intestinal flora when oral stimulation with IEC ameliorates *S.Typhimurium*-induced pathological injury data is available here: https://figshare.com/s/8ad0acce0db9a0f3f96e, DOI:10.6084/m9.figshare.21529674.

## Ethics statement

The animal study was reviewed and approved by the ethics review committee of Experimental Animal, Northwest A&F University (Approval Number.NWAFC 1008).

## Author contributions

XX helped MC design this study and critically revised the manuscript. MC performed the experiments and statistical analysis and wrote the first draft of the manuscript. GT, FY, SW, and XW participated in the experiment for sampling. JY gave valuable suggestions on the experiment design and the discussion of the results. All authors contributed to the article and approved the submitted version.
